# Secure Data Aggregation with Fully Homomorphic Encryption in Large-Scale Wireless Sensor Networks

**DOI:** 10.3390/s150715952

**Published:** 2015-07-03

**Authors:** Xing Li, Dexin Chen, Chunyan Li, Liangmin Wang

**Affiliations:** 1 School of Computer Science and Communication Engineering, Jiangsu University, Zhenjiang 212013, China; E-Mails: lixing0409@163.com (X.L.); lcy20110416@163.com (C.L.); 2 College of Computer Science, Sichuan University, Chengdu 610065, China; E-Mail: lhy5154@163.com

**Keywords:** wireless sensor networks, data aggregation, fully homomorphic encryption, data integrity, message authentication code

## Abstract

With the rapid development of wireless communication technology, sensor technology, information acquisition and processing technology, sensor networks will finally have a deep influence on all aspects of people’s lives. The battery resources of sensor nodes should be managed efficiently in order to prolong network lifetime in large-scale wireless sensor networks (LWSNs). Data aggregation represents an important method to remove redundancy as well as unnecessary data transmission and hence cut down the energy used in communication. As sensor nodes are deployed in hostile environments, the security of the sensitive information such as confidentiality and integrity should be considered. This paper proposes Fully homomorphic Encryption based Secure data Aggregation (FESA) in LWSNs which can protect end-to-end data confidentiality and support arbitrary aggregation operations over encrypted data. In addition, by utilizing message authentication codes (MACs), this scheme can also verify data integrity during data aggregation and forwarding processes so that false data can be detected as early as possible. Although the FHE increase the computation overhead due to its large public key size, simulation results show that it is implementable in LWSNs and performs well. Compared with other protocols, the transmitted data and network overhead are reduced in our scheme.

## 1. Introduction

Wireless sensor networks (WSNs), which can have hundreds or thousands of sensor nodes deployed over a monitored region, are being increasingly used in several applications such as military reconnaissance, target tracking, environmental monitoring, and medical monitoring [1,2,3,4]. In these large-scale wireless sensor networks (LWSNs), the amount of data processed is very large and the data redundancy is very high, but the sensing devices (nodes) in LWSNs have limited computing power and communication resources, leading to network performance degradation or collapse once the nodes’ limited power is depleted [5]. As a result, energy saving technologies must be considered. In-network data aggregation can reduce communication overhead and hence reduce the energy consumption, especially in LWSNs.

Since many sensor nodes are deployed in hostile environments, data aggregation faces many security issues such as data confidentiality and integrity [6], and various attacks like selective forwarding attack, replay attack, false data injection *etc.* [7,8,9]. When sensor nodes are compromised, it is easy for the adversary to change the aggregation result and inject false data into the LWSNs. Hence, data aggregation protocols in LWSNs should be designed with security in mind while achieving energy efficiency, even in the presence of malicious nodes in networks.

The standard method to preserve confidentiality is to encrypt the data. Secure data aggregation protocols can be categorized as hop-by-hop encryption and end-to-end encryption [10,11]. The hop-by-hop secure data aggregation protocols cannot provide data confidentiality at aggregators because the aggregators are required to share keys with their neighboring nodes [8,9]. In end-to-end secure data aggregation protocols, intermediate nodes aggregate data directly without decrypting the received data. When they are captured, an adversary cannot get the original information. The most common method, named privacy homomorphic cryptography, has been studied for data aggregation in WSNs to achieve end-to-end confidentiality [12,13,14,15]. However, this method only deals with limited arithmetic operations on ciphertexts because the noise contained in ciphertexts would become larger with successive homomorphic multiplications and make the decryption fail. An optimized method which can evaluate any function on encrypted data is fully homomorphic encryption (FHE) [16,17,18]. To the best of our knowledge, this is the first paper that combines the concept of FHE with secure data aggregation instead of using privacy homomorphism in WSNs. Confidentiality itself is not enough since an adversary is still able to add some fragments or falsify content to change the data although it knows nothing about the data. For this reason, Message Authentication Code (MAC) protocols are often used to detect false data and protect data integrity [14,15]. However, these protocols only detect the false data in BS, which might lead to large communication overhead.

To solve the problems mentioned above, this paper presents a secure data aggregation with fully homomorphic encryption for LWSNs. Our scheme can provide false data detection and secure data aggregation against up to *T* compromised sensor nodes. In particular, the main contributions of this paper may be summarized as follows:

- To address the drawbacks of privacy homomorphic cryptography, we focus on the investigation of achievable FHE for end-to-end data confidentiality in LWSNs. The designed FESA can be implemented in sensor nodes, by which aggregators can do unlimited arithmetic aggregation functions on ciphertexts.
- In order to detect false data during both data forwarding and aggregation processes as early as possible, we propose MFN-group network structure which consists of monitoring node, forwarding node and neighboring node of aggregator. In this structure, the forwarding node and neighboring node verify the data computed by the monitoring node in the same group, and detect false data when it appears immediately. Thereby, this structure reduces the data transmission in the network with compromised nodes.
- We apply MACs to protect data integrity confidentially and conveniently. In our scheme, monitoring nodes compute MACs for the aggregated data, so that the group members can verify the integrity of data through computing and comparing the MACs directly. Therefore, we do not have to forward the plaintext for the verification. If the aggregator is captured, the attacker will not know the data.

The rest of the article is organized as follows: in Section 2, we overview some related works on secure data aggregation. Section 3 introduces the network model and background knowledge. In Section 4, we give the detailed descriptions of our scheme FESA. Section 5 analyzes the performance of security properties. The simulation results of the proposed schemes are presented in Section 6. Finally, we summarize our conclusions in Section 7.

## 2. Related Works

Data aggregation aims to combine and summarize data packets of several sensor nodes so that amount of data transmission is reduced. It enhances the network lifetime because data transmission accounts for 70% of the energy cost of computation and communication [19]. While increasing the network lifetime, data aggregation may negatively affect other performance metrics such as delay, accuracy, fault-tolerance, and security. Hence, it is a challenging task to provide efficient secure data aggregation. The basic method to protect data confidentiality is encryption. The pairwise key establishment protocol proposed in [20] using direct key establishment mechanism for neighboring nodes and path key establishment mechanism for multi-hop nodes. Group key establishment scheme [21] allows a key generation center to broadcast group key information to all group members at one-time, and only authenticated group member can retrieve the group key. However, it usually applied for small scale WSNs. Another existing group key establishment scheme [22] can be implemented in large scale networks, and incurs only *O*(*n*) communication overhead when establishing group keys.

A witness-based data aggregation scheme for WSNs is proposed in [23]. The witness nodes of each aggregator also perform data aggregation and compute MACs of the aggregated data. The aggregator collects and forwards the MACs which are sent by witness nodes to the BS. Those MACs are used to verify the correctness of the data aggregated by aggregators. This protocol offers only integrity property to the data aggregation security.

In [8], data are encrypted hop-by-hop, and the algorithm forms pairs of sensor nodes such that one computes a MAC and the other one verifies it. In this scheme, data aggregation is not allowed if it requires alterations in the data, and it was improved by Ozdemir and Cam in [9]. The DAA protocol in [9] integrates false data detection with data aggregation and confidentiality. The monitoring nodes of aggregator also perform data aggregation and compute the MACs for data verification. In these protocols, the computation overhead is increased for hop-by-hop encryption.

In order to mitigate the drawbacks of the hop-by-hop schemes, some end-to-end protocols are proposed. The mechanism described in [13], based on homomorphic hash and identity-based aggregate signatures, lets the BS and each node share a different key, and then uses weights to verify the authenticity of the aggregated data. Elliptic curve cryptography-based homomorphic encryption is used in [14] to achieve hierarchical data aggregation, data integrity and confidentiality. This scheme is based on asymmetric cryptography which has a larger computation overhead. A secure data aggregation scheme based on homomorphic primitives is proposed in [15]. It applies symmetric cryptography-based privacy homomorphism and homomorphic MAC to protecting data confidentiality and detecting data integrity. It also computes all packets during the process of integrity verification. The comparison of those secure data aggregation protocols is presented in Table 1.

**Table 1:** The comparison of secure data aggregation protocols with respect to WSNs security requirements.

Parameters	Data Confidentiality	Encryption Method	Data Integrity	Integrity Detection Method	Integrity Detection Position
Du *et al.* [23]	×	–	√	MAC	Data aggregation
DAA [9]	√	Hop-by-hop symmetric	√	MAC	Data aggregation, forwarding and BS
CDA [12]	√	End-to-end symmetric	×	–	–
Niu *et al.* [13]	√	End-to-end symmetric	√	Homomorphic hash	Data forwarding and BS
IPHCDA [14]	√	End-to-end asymmetric	√	MAC	BS
SDA-PH [15]	√	End-to-end symmetric	√	MAC	BS

The most common method to achieve end-to-end confidentiality is privacy homomorphic cryptography. However, this method only deals with limited arithmetic operations on ciphertexts. The optimized method which can evaluate unlimited arithmetic aggregation function on encrypted data is FHE [16,17,18]. FHE originally called a privacy homomorphism [16,17], was introduced by Rivest, Adleman and Dertouzous shortly after the invention of RSA [24]. The first construction of an FHE (based on ideal lattices) was described by Gentry in [17]. Currently, research on FHE is mostly concentrated on improving the FHE algorithm, however, its applications are studied rarely and have only investigated its use in the cloud computing environment [25].

## 3. Model and Background

We now present the network assumptions, discuss the attack model, and formally state the problem that we address in this paper. A list of notations used in this paper is given in Table 2.

**Table 2:** Notations used in FESA, where the value of *T* depends on security requirements, node density, packet size, and the amount of tolerable overhead.

Notation	Meaning
*T*	Network security factor
*A_b_*	Backward data aggregator
*A_c_*	Current data aggregator
*A_n_*	Next data aggregator
*N_i_*	Neighboring node *i* of aggregator
*F_j_*	Forwarding node *j* of aggregator
*M_l_*	Monitoring node *l* of aggregator
*K_group_*	Group key of MFN-group
FH(*X*)	Fully homomorphic value of node *X*
subMAC(FH(*X*))	subMAC of node *X*’s fully homomorphic value

### 3.1. Network Assumptions

In this paper, we consider a large-scale sensor network with densely deployed sensor nodes. We assume a general multi-hop network with a set *S* = {*s*_1_, …, *s_d_*} of *d* sensor nodes and a single trusted BS which has a long-lasting power. Some sensor nodes are dynamically assigned as aggregators based on their remainder energy levels to aggregate data from their neighboring nodes. The sensor network is mostly static with a topology known to BS. Typical aggregation functions include SUM, AVERAGE, COUNT, MAX, and MIN, and all of them can be reduced to the additive aggregation function SUM [26].

### 3.2. Attack Model

A malicious attacker can launch a wide variety of attacks to break the privacy and integrity of aggregation results. In general, it is impossible to prevent all kinds of attacks. This paper only considers the following categories of attacks in WSNs:

- Eavesdropping: It is the most common and easiest form of attack on data confidentiality. An attacker attempts to obtain private information by overhearing the transmissions over its neighboring wireless links. We assume the attacker can eavesdrop on the entire network.
- False data injection: This can possibly occur during data aggregation or data forwarding. A compromised node can distort data integrity by injecting false data and then drain the limited energy resources of the network. A joint data aggregation and false data detection technique has to ensure that data are changed by data aggregation only.
- Sybil attack: It is a type of attacks where the attacker is able to present more than one identity within the network. An adversary can launch a Sybil attack and generate *n* or more witness identities to make the base station accept the aggregation results.

### 3.3. Fully Homomorphic Encryption

FHE is a powerful technique that allows aggregators to aggregate received data directly without decryption [16,17,18]. At a high-level, the essence of FHE is simple: given ciphertexts that encrypt *π*_1_, …, *π_t_*, FHE should allow anyone (not just the key-holder) to output a ciphertext that encrypts *f*(*π*_1_, …, *π_t_*) for any desired function *f*, as long as that function can be efficiently computed.

The first construction of an FHE was conducted in several steps by Gentry [17]. First, one constructs a somewhat homomorphic encryption (SWHE) scheme, which only supports a limited number of multiplications: ciphertexts contain some noise that becomes larger with successive homomorphic multiplications, and only ciphertexts whose noise size remains below a certain threshold can be decrypted correctly. The second step is to squash the decryption procedure associated with an arbitrary ciphertext so that it can be expressed as a low degree polynomial in the secret key bits. Then, Gentry’s key idea, called bootstrapping, consists in homomorphically evaluating this decryption polynomial on encryptions of the secret key bits, resulting in a different ciphertext associated with the same plaintext, but with possibly reduced noise. This refreshed ciphertext can then be used in subsequent homomorphic operations. By repeatedly refreshing ciphertexts, the number of homomorphic operations becomes unlimited, resulting in a FHE scheme.

In [18], Dijk using the elementary modular arithmetic to construct a simple and secure SWHE scheme, based on the idea of Gentry in [17], to convert it to an FHE scheme over the integers, called DGHV scheme. This scheme merely uses addition and multiplication over the integers and its concepts are simple. An FHE scheme has four algorithms: KeyGen, Encrypt, Decrypt, and an additional algorithm Evaluate. The algorithm Evaluate takes as input a public key *pk*, a circuit *C*, a tuple of ciphertexts ***c*** < *c*_1_, …, *c_d_*> (one for every input bit of *C*), and outputs another ciphertext *c*. In the DGHV scheme, because computing the decryption seems to require Boolean circuits that are deeper (by a constant factor) than what the SWHE scheme can handle, the “squash the decryption circuit” is used for transformation. In this transformation, some extra information about the secret key are added to the public key, and then used to “postprocess” the ciphertext. The post-processed ciphertext can be decrypted more efficiently than the original ciphertext, thus making the scheme bootstrappable. In this paper, we apply the DGHV scheme to protect end-to-end data confidentiality.

### 3.4. Message Authentication Code

**Figure 1:**
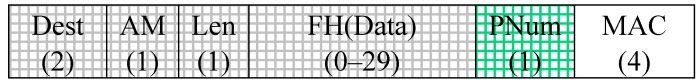
The packet structure of FESA scheme, where MAC is composed of *T* + 1 subMACs. The byte size of each field is enclosed in parentheses. The acronyms of the fields are destination address (Dest), active message type (AM), message length (Len), packet sequence number (PNum).

MACs are used by nodes to verify the data integrity. The data packet structure in this paper is the link layer data packet structure under TinySec authentication mode [27], which uses a MAC field to verify the whole packet data. The data stored in FH(Data) is encrypted by privacy homomorphic encryption. Let Dest, AM, Len and PNum denote the destination address, active message type, message length and packet sequence number, respectively. The packet structure of our scheme is shown in Figure 1.

In this structure, the 4-byte MAC consists of *T* + 1 subMACs, and each of these is constructed by selecting some bits of a MAC, such that one of them is computed by an aggregator and the remaining *T* subMACs are computed by its *T* monitoring nodes. Algorithm 1 shows the generation of the subMAC which is in the similar approach with [9]. We assume that each sensor node has the same pseudo-random number generator (PRNG) [28] that generates random numbers ranging from 1 to 32. After the groups are formed and their group keys (*K_group_*) are established, sensor nodes initiate their PRNGs using their *K_group_* as the seed. Let *Z* denotes the total bits of the MAC, each random number indicates the index of a bit location in MAC. To select the bits from MAC, the monitoring node *M_l_* runs its PRNG *Z*/(*T* + 1) times, results in *Z*/(*T* + 1) random numbers, and then forms the subMAC. The subMAC computed by *M_l_* can be verified by the corresponding group member *F_j_* who computes the subMAC and matches each other. Note that PRNGs can be out of synchronization due to packet loses. In this paper, PRNG synchronization is achieved using packet sequence numbers that are added to aggregated data packets [9].

**Algorithm 1** The generation of subMAC**Assumption:** *M_l_* is a member of a node group, and it shares the key *K_group_* with other group mates. Each sensor node has the same pseudo-random sequence generator.
**begin** 
 node *M_l_* uses *K_group_* to compute MAC(*M_l_*); 
 *M_l_* runs pseudo-random sequence generator *Z*/(*T* + 1) times to select *Z*/(*T* + 1) bits of MAC(*M_l_*), and then forms the subMAC(*M_l_*);
 return subMAC(*M_l_*);
**end**

In the formation of MACs, the aggregator determines the order of subMACs in any way and informs each group members of monitoring node about its subMAC location individually. Consequently, an adversary cannot know in advance the exact location of subMAC bits for a given forward node. Therefore, in order to inject a false message, an adversary has to try all possibilities, which improves the security of the network to a large extent.

## 4. FESA: Secure Data Aggregation with Fully Homomorphic Encryption in Large-Scale Wireless Sensor Networks

In this section, we present the detailed structure and design of our scheme.

### 4.1. Network Structure

**Figure 2:**
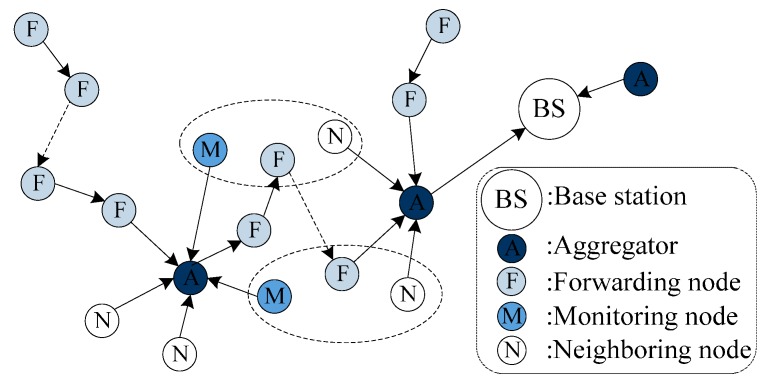
The network structure of FESA, where the three nodes within a dotted line are in the same group and share one group key.

As is shown in Figure 2, the network structure is improved by forming MFN-group based on [9]. The formation of network structure is shown in Algorithm 2, details are as follows.

**Algorithm 2** The formation of network structure**Assumption:** Sensor nodes are densely distributed in the network.
**begin**
**Step1.** Use SANE protocol to select the aggregation nodes, make    sure at least *T* nodes between two consecutive aggregation nodes, and each aggregation node has at least *T* neighboring nodes.
**Step2.** Use MNS algorithm to select *T* monitoring nodes for each aggregation node.
**Step3.** 1: *A_n_* computes the MAC(*N_i_*) of its neighboring nodes *N_i_* and transmits the messages to *A_c_* with the list message of *N_i_* via *F_j_*.
       2: *F_j_* adds its own ID into the message while transmitting.
       3: *A_c_* concatenates the IDs of *F_j_* and *N_i_* respectively in a random order and indexes them 1 to *h* and 1 to *s*. Then *A_c_* computes the MAC of the concatenated IDs and broadcasts MAC, *h* and *s* to *M_l_*.
       4: *M_l_* selects an index number between 1 to *h* and 1 to *s*, and knows the group mates *F_j_* 
**end**

#### 4.1.1. Selection of Aggregators

We run the secure data aggregator selection protocol (SANE) [29] to select data aggregators which satisfy two conditions:

(1) There are at least *T* nodes, called forwarding nodes, on the path between any two consecutive aggregators.
(2) Each aggregator has at least *T* neighboring nodes.

#### 4.1.2. Selection of Monitoring Nodes

In order to perform secure data aggregation, each aggregator is monitored by its *T* neighboring nodes out of a total of *s* neighboring nodes, for *s* ≥ *T*. The monitoring nodes are selected by the Monitoring Node Selection (MNS) algorithm [9]. The basic idea of this algorithm is to assign indices to the neighboring nodes in some order and then compute *T* indices by applying modulus operation to the sum of some random numbers generated by the neighboring nodes. Any neighboring node whose index is equal to one of these *T* indices becomes a monitoring node. The advantage of this algorithm is that, aggregator and all neighboring nodes are involved with the selection of monitoring nodes, part of nodes compromised cannot damage the selection result, which can minimize the adverse impact of a compromised node.

#### 4.1.3. Formation of MFN-Groups

**Figure 3:**
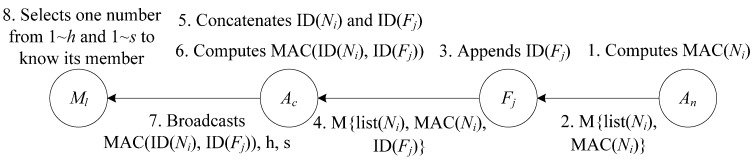
The message transmission process in the formation of MFN-group.

In the network of this scheme there are *T* MFN-groups formed, each of which consists of a monitoring node of *A_c_*, a forwarding node of *A_c_* and a neighboring node of *A_n_*. It is assumed that a monitoring node can establish a group key *K_group_* with its members that are multiple hops away using an existing group key establishment scheme such as [22].We assume that a path already exists between any two consecutive aggregators via forwarding nodes, and that each aggregator uses only one outgoing path towards BS at a given time. These two consecutive aggregators form one pair and share a symmetric key for false data detection. If they do not have a shared key, they establish a symmetric key *K_pair_* using an existing pairwise key establishment algorithm such as direct key establishment method [20]. The message transmission process in the formation of MFN-group is showed in Figure 3. The formation process of MFN-groups includes the following steps:

(1) *A_n_* computes the MAC of its neighboring node list, adds this MAC and its neighboring node list into a group discovery message M.
(2) Then *A_n_* sends M to *A_c_* via forwarding nodes on the path between *A_c_* and *A_n_*.
(3) Each forwarding node appends its ID to the message it forwards.
(4) Assuming that *A_n_* has *s* neighboring nodes and there are *h* forwarding nodes between *A_c_* and *A_n_* (*s*, *h* ≥ *T*), then the message *A_c_* received contains the IDs of its forwarding nodes and neighboring nodes of *A_n_*.
(5) After receiving this message, *A_c_* concatenates the IDs of the forwarding nodes in a random order and indexes them 1 to *h*, and also concatenates the IDs of the neighboring nodes in a random order and indexes them 1 to *s*.
(6) Then, *A_c_* computes the MAC of the concatenated IDs list using *K_group_*.
(7) *A_c_* broadcasts the MAC, *h* and *s* to its *T* monitoring nodes.
(8) Each monitoring node of *A_c_* selects an index number between 1 to *h* and 1 to *s*, so that *M_l_* of *A_c_* knows its group members from the concatenated IDs list it received.

In this process, the monitoring nodes of *A_c_* verify the correctness of the broadcasted IDs and their indexed orders using the previously broadcasted MAC of the concatenated IDs list. Therefore, the formation of MFN-groups cannot be affected even if *A_c_* is attacked or damaged, and this process makes sure that each monitoring node corresponds to only one forwarding node and one neighboring node.

In conclusion, as shown in Algorithm 2, the process of forming the network structure in this scheme can finally form the MFN-groups. After the monitoring node of *A_c_* performs data aggregation and computes subMAC for the aggregated data, the characteristic of such network structure ensures that the corresponding forwarding node of *A_c_* can verify the data integrity during data transmission, and the corresponding neighboring node of *A_n_* can verify the data integrity during data aggregation. Therefore, it can also detect any false data injection during data transmission and data aggregation.

### 4.2. Key Generation

After the formation of network structure, we use the DGHV scheme to encrypt the source sensor data, evaluate the encrypted data and decrypt the aggregated encrypted data. The parameters (all polynomial in the security parameter *λ*) used in our scheme are as follows: *γ* is the bit-length of the integers in the public key, *η* is the bit-length of the secret key (which is the hidden approximate-gcd of all the public-key integers), *ρ* is the bit-length of the noise (*i.e.*, the distance between the public key elements and the nearest multiples of the secret key), and *τ* is the number of integers in the public key. Let *κ =* *γη/ρ*’, *θ* = *λ*, and *Θ* = *ω*(*κ∙*log *λ*). For a secret key sk* = *p* and public key pk* from the original SWHE scheme ε*, we add to the public key a set ***y*** = {*y*1,...,*yΘ*} of rational numbers in [0,2) with *κ* bits of precision, such that there is a sparse subset *S*⊂{1,..., *Θ*} of size *θ* with $\;\underset{\text{i}\in \text{S}}{\sum }{\text{y}}_{\text{i}}\approx \text{1/p}\left(\text{mod 2}\right)$. We also replace the secret key by the indicator vector of the subset *S*.

For the key generation phase, BS respectively generates the secret key *sk* and public key *pk*as follows:

*sk* = ***s*** (1)

*pk* = (pk*, ***y***) (2)

Then BS informs the source nodes the public key *pk* for them to encrypt the plaintexts which are sensed by the source nodes.

### 4.3. Data Encryption

Choose a random subset *S*⊆{1,2,...,*τ*} and a random integer *r* in (−2*^ρ^’*, 2*^ρ^’*), *m* ∈ {0,1}.Then, each source sensor node picks the source data, and utilizes *pk* to generate a ciphertext as follows:

(3) $$\text{ c}\leftarrow {\left[\text{m+2r+2}\underset{\text{i}\in \text{S}}{\sum }{\text{x}}_{\text{i}}\right]}_{{\text{x}}_{\text{0}}}$$

For the public key, sample $\;{\text{x}}_{\text{i}}\overset{\text{\$}}{\leftarrow }{\text{D}}_{\text{γ,ρ}}\left(\text{p}\right)$ for i= 0,…,τ, where:

(4) $${\text{D}}_{\text{γ,ρ}}\left(\text{p}\right)\text{ = }\left\{\text{choose q}\overset{\text{\$}}{\leftarrow }\text{Z}⋂[\text{0}{\text{,2}}^{\text{γ}}\text{/p), r}\overset{\text{\$}}{\leftarrow }\text{Z}⋂\left({\text{-2}}^{\text{ρ}}{\text{,2}}^{\text{ρ}}\right)\text{ : output x = pq + r}\right\}$$

After that, this ciphertext *c* is forwarded to the next forwarding node or aggregator to process.

### 4.4. Data Aggregation and Integrity Detection

When data are transferred to an aggregator, the aggregator can aggregate the data directly for the property of the FHE scheme. Our scheme merely processes addition and multiplication over the integers. Given the (binary) circuit *Cε* with *d* inputs, and *d* ciphertexts *ci*, apply the (integer) addition and multiplication gates of *Cε* to the ciphertexts, performing all the operations over the integers, and return the resulting integer c* which satisfies ${\text{Decrypt}}_{\text{ε}}\left({\text{sk, c}}^{\text{*}}\right)\text{ = C}\left({\text{m}}_{\text{1}}{\text{, …, m}}_{\text{d}}\right)$:

(5) $${\text{c}}^{\text{*}}{\text{ = Evaluate}}_{\text{ε}}\left({\text{pk, C, c}}_{\text{1}}{\text{, …, c}}_{\text{d}}\right)$$

After generating the ciphertext c*, then for *i* ∈{1,..., *Θ*}, set

*z_i_* ←[c*·*y_i_*]_2_ (6)

In order to achieve data confidentiality, data integrity and false data detection during data aggregation and data forwarding, we propose a data aggregation and integrity detection algorithm to verify the data integrity and detect any false data injections in the whole network. The basic idea of the data aggregation and integrity detection algorithm is that a monitoring node computes the subMAC of the aggregated fully homomorphic value, and the corresponding forwarding node and neighboring node in the same group verify this subMAC. When data reaches the neighboring node of *A_c_*, by calculating the subMAC of the received fully homomorphic value, the neighboring node verifies the integrity during data aggregation which is done by the backward aggregator, therefore enabling the detection of data integrity in the data aggregation process. When data reaches the forwarding node of *A_c_*, by calculating the subMAC of the received fully homomorphic value, the forwarding node verifies the integrity during the data transmission process from *A_c_* to this forwarding node, therefore enabling the detection of data integrity in the data transmission process. The detailed process of data aggregation and integrity detection algorithm is shown in Algorithm 3. The general process of this algorithm can be described as follows:

(1) The current aggregator *A_c_* first collects data from its neighboring nodes. In order to detect whether any false data exists in the backward aggregator *A_bq_*, *A_c_* broadcasts the data it received to its neighboring nodes, such that the neighboring node of *A_c_* who is in the same MFN-group with the monitoring node of *A_bq_* verifies the data integrity by computing the subMAC. If there is at least one verification fail, *A_c_* discards this data and informs *A_bq_*.
(2) Aggregator *A_c_* and its *T* monitoring nodes aggregate the received data, respectively. *A_c_* computes the subMAC of aggregated data using *K_pair_*, monitoring node *M_l_* computes the subMAC of aggregated data using *K_group_*, then finally *T* + 1 subMACs are obtained.

**Algorithm 3** Data aggregation and integrity detection algorithm**Assumption:** *A_c_* has *p* leaf aggregation nodes {*A_b_*_1,_ …, *A_bp_*}, *s* neighboring nodes {*N*_1_, …, *N_s_*}, *T* monitoring nodes {*M*_1_, …, *M_T_*} and *h* forwarding nodes {*F*_1_, …, *F_h_*}.
**begin**
**Step 1.** *A_c_*and *N_i _* verify the data integrity of *A_bq_* during data aggregation.
**for** (*q* =1 to *p*) **do**
   1: The last forwarding node *N_i_* of *A_bq_*receives the data sent by *A_bq_*, then transmits it to *A_c_*
   2: *A_c_*receives the data and computes subMAC(FH(*A_bq_*)) to verify the data integrity
   3: **if** the subMAC(FH(*A_bq_*)) computed by *A_c_* and *A_bq_* are not equal
**then** *A_c_* drops the data immediately and informs *A_bq_*	
   4: **else** *A_c_* broadcasts the data packet to *N_i_*
   5: All neighboring nodes of *A_c_*, which are the member of MFN-groups, verify the data integrity.
   6: **if** the subMAC computed by *N_i_* is not equals to which computed by the same MFN-group member monitoring nodes of *A_bq_*
	**then** *A_c_*drops the data immediately and informs *A_bq_*
  **end for**
**Step 2.** Aggregate the data.
    1: *A_c_* and each monitoring node *M_l_* aggregate all the verified data it received (*l* = 1 to *T*).
    2: *A_c_* uses the key it shared with *A_n_* to compute subMAC(FH(*A_c_*)). *M_l_* uses the group key to compute subMAC(FH(*M_l_*)). Each *M_l_* sends its subMAC(FH(*M_l_*)) to *A_c_*.
    3: *A_c_* forms a packet containing FH(*A_c_*), subMAC(FH(*A_c_*)) and subMAC(FH(*M_l_*)) and sends it to its first forwarding node.
**Step 3.** The forwarding nodes of *A_c_* verify the integrity of the data during data transmission.	
   **for** (*j* = 1 to *h*) **do**
    1: **if** *F_j_* is not the member of MFN-groups
      **then** *F_j_* just transmits the packet it received to the next forwarding node or *A_n_*
	2: **else** *F_j_* verifies the subMAC(FH(*M_l_*))	
    3: **if** the verification is successful
	  **then** *F_j_* transmits the packet to the next forwarding node or *A_n_*
	4: **else** *F_j_* drops the packet and informs *A_c_* about it
      **end for**
**Step 4.** Repeat the steps above, until the packet arrives at the base station.
     Relabel *A_c_* and *A_n_* as *A_b_*and *A_c_*, respectively. Go to **Step 1** to repeat the steps, until the packet is transmitted to the base station.
**end**

(3) *A_c_* collects these *T* + 1 subMACs to transmit to the next aggregator *A_n_* along with aggregated data via forwarding nodes. When the forwarding nodes of *A_c_* receive the packet, the node which is the group member of MFN-group, expressed as *F_j_*, computes the subMAC of the aggregated data which is aggregated by its corresponding monitoring node, and then matches this subMAC. If the verification fails, *F_j_* discards this packet immediately and informs *A_c_*. Otherwise, if verification is successful, *F_j_* forwards this packet.
(4) When the packet arrives at the next aggregator *A_n_*, *A_n_* verifies the subMAC which is calculated by *A_c_*. If this verification is successful, *A_c_* and *A_n_* are relabeled as *A_bq_* and *A_c_*, respectively, then repeat step 1 to verify the data integrity during data aggregation.

### 4.5. Data Decryption

When the data are arrived at BS, the final result is obtained by the following equation. From [22], we know that the result can be correctly decrypted:

(7) $$m\prime \leftarrow {\left[c^{*}-\left\lfloor \sum_{i}S_{i}Z_{i}\right\rceil \right]}_{2}$$

## 5. Security Analysis

In this section, we analyze the performance of FESA scheme in terms of security properties including data confidentiality and integrity.

### 5.1. Data Confidentiality

In WSNs, data confidentiality ensures that important data will never be disclosed to an unauthorized third party. This scheme achieves data confidentiality in the following parts.

#### 5.1.1. Security Analysis of FHE

This paper uses a FHE scheme to achieve end-to-end data confidentiality. Data are encrypted in source nodes and decrypted in BS using the FHE scheme. During the data forwarding and aggregation processes, the intermediate nodes cannot decrypt the encrypted data packets without knowing the key, so it is hard for an adversary to breach all encryption keys or find the plaintext if it knows only the ciphertext. Therefore, our scheme is secure to ciphertext-only attacks. Even if the aggregation data is disclosed, the adversary can only get the aggregation result but not sensor data. Similarly, it is also secure to plaintext-only attacks.

#### 5.1.2. Security Analysis of SubMAC

In our scheme, the data packet reserves 4 bytes for MAC. The security of a 4-byte MAC is quantified as 2^4×8^ because an adversary has a 1 in 2^4×8^ chance in blindly forging the MAC. While increasing the size of MAC is also increasing the communication overhead, the subMAC which has the size of 32/(*T* + 1) bits is employed in this scheme, and *T* + 1 subMACs computed by *T* + 1 nodes form a MAC. Hence, an adversary can successfully forge a valid MAC if it finds all *T* + 1 subMACs with the probability of 1 in 2^32/(*T*+1)^ for each subMAC. Thus, the probability that the false data are not detected by the MAC is (1/2^32/(*T* +1)^)*^T^*^+1^ = 1/2^32^.

### 5.2. Data Integrity

Data integrity guarantees that data or information being transferred is never corrupted during data transmission and storage process. Although data confidentiality guarantees that only intended parties obtain the un-encrypted plain data, it does not protect data from being altered. Given the RSA encryption of numerical information as an example, a hacker or malicious user can do linear operation on the ciphertext and change the value even without breaking the key. When a sensor node is captured, the intruder is assumed to access all the available security information, such as cryptographic keys. Two conclusions can be put forth as two lemmas:

**Lemma 1.** *Assume that A_c_ is compromised and there are additional at most T − 1 collaborating compromised nodes among the neighboring nodes of A_c_ and A_n_. Then, any false data injected by A_c_ are detected by the A_n_’s neighboring nodes only*.
**Proof of Lemma 1.** The neighboring nodes of *A_c_* verify all the data broadcasted by *A_c_*, each monitoring node of *A_c_* also aggregates the entire data by itself and then computes a subMAC for the aggregated data. If *A_c_* injects false data, it can be detected by *A_n_*’s neighboring nodes that are the MFN-group members of the monitoring nodes of *A_c_*. Since the subMACs of the plain aggregated data are verified by *T* neighboring nodes of *A_n_*, *A_c_* needs at least *T* compromised monitoring nodes to inject false data.
**Lemma 2.** *Assume that A_c_ and A_n_ are not compromised, even if all forwarding nodes of A_c_ are compromised, false data that they inject are detected by A_n_*.
**Proof of Lemma 2.** Those forwarding nodes that are the MFN-group members of *A_c_*’s monitoring nodes verify the transferred data, but they do not compute new subMACs for the verified data. Thus, an attacked forwarding node that injects false data cannot add a new subMAC for its false data. Because all the forwarding nodes of *A_c_* are assumed to be compromised, the false data injected by these compromised nodes are not detected during data forwarding. This implies that *A_n_* receives the false data. In Algorithm 3, aggregators verify the received data using the subMACs computed by the backward aggregators. Therefore, when *A_n_* receives the false data from compromised forwarding nodes, *A_n_* fails to verify the subMAC computed by *A_c_*, which is assumed to be not compromised.

## 6. Simulation Results

The simulation was run on a PC with Core i3-3220 CPU, 4G memory, and Win7 OS. The simulation is implemented in the ns-2 simulator [30]. In order to make the comparison fair, the simulation parameters we used which are listed in Table 3 are similar to those of the DAA [9] and SDA-PH [15] schemes. Some nodes are designed as aggregators. The BS is located in the central area. Data are assumed to be generated mainly by the nodes located at the edges of the network, although any node is allowed to sense events and generate data. To show the benefit of our scheme, we evaluate the computational and communication overhead of FESA, DAA and SDA-PH schemes.

**Table 3:** Simulation parameters.

Parameters	Value
Number of nodes	250
Terrain dimensions	400 × 400 m^2^
Transmission range	50 m
Data rate	0.1 Mbps
Propagation pathloss model	Two-ray
MAC protocol	IEEE 802.11
Routing protocol	DSDV
Simulation time	200 s

### 6.1. Computational Overhead

**Figure 4:**
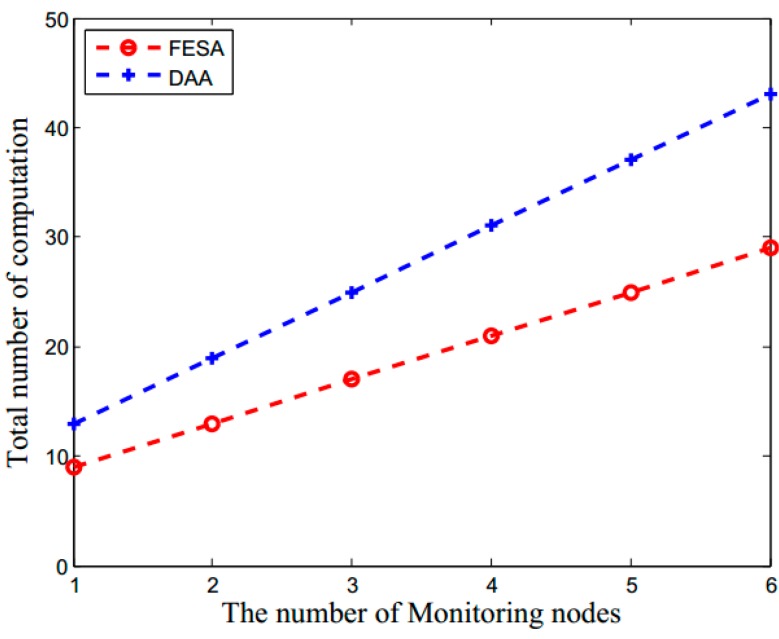
The total number of computations (which includes the computations of encryption, decryption and MACs) *versus* the number of network monitoring nodes for FESA and DAA.

The energy consumption in LWSNs is mainly due to data transmission. Thus, it is particularly important to reduce data redundancy and detect false data as early as possible. The energy consumption in this scheme mainly includes the computational overhead of encryption and decryption, the computational overhead of MACs and data communication overhead. The total number of computations in this scheme is shown in Figure 4, which is affected by the number of monitoring nodes and contains the computation of encryption, decryption and MACs. As shown in Figure 4, as the monitoring nodes increase, the total number of computations becomes larger. Since the DAA needs to encrypt and decrypt hop-by-hop, its number of computations is more than FESA.

#### 6.1.1. Computational Overhead of Encryption and Decryption

The characteristics of end-to-end encryption protocols and hop-by-hop encryption protocols indicate that the latter one increase the encryption and decryption operations in aggregators. DAA needs *T* + 2 encryption and decryption processing in the intermediate nodes [9]. A comparison shows that the computational overhead of data confidentiality decreases while protecting data integrity.

#### 6.1.2. Computational Overhead of MACs

In FESA, each aggregator and its monitoring nodes need to compute *T* + 1 subMACs. Because each subMAC is obtained by first computing a MAC and then selecting some bits of it, forming *T* + 1 subMACs requires the computation of *T* + 1 MACs. Moreover, additional 2*T* + 1 MAC computations are needed to verify all the subMACs by forwarding nodes of *A_c_*, *A_c_*, and neighboring nodes of *A_n_*. Hence, FESA totally needs 3*T* + 2 MAC computations and *T* + 1 aggregation processes during the whole verification between two consecutive aggregators, while DAA needs total 4 × (*T* + 1) MAC computations and *T* + 1 aggregation processes. The comparison is showed in Table 4.

**Table 4:** Comparison for computational overhead of MACs.

Position	Aggregators	Monitoring Nodes	Forwarding Nodes	Neighboring Nodes
DAA	4	2*T*	*T*	*T*
FESA	2	*T*	*T*	*T*

### 6.2. Communication Overhead

The main communication overhead of FESA scheme is the MAC transmission for data transmission and false data detection during data aggregation.

#### 6.2.1. Communication Overhead in Aggregator

**Figure 5:**
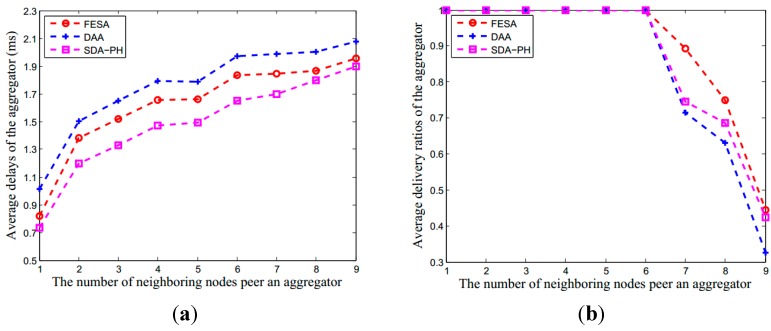

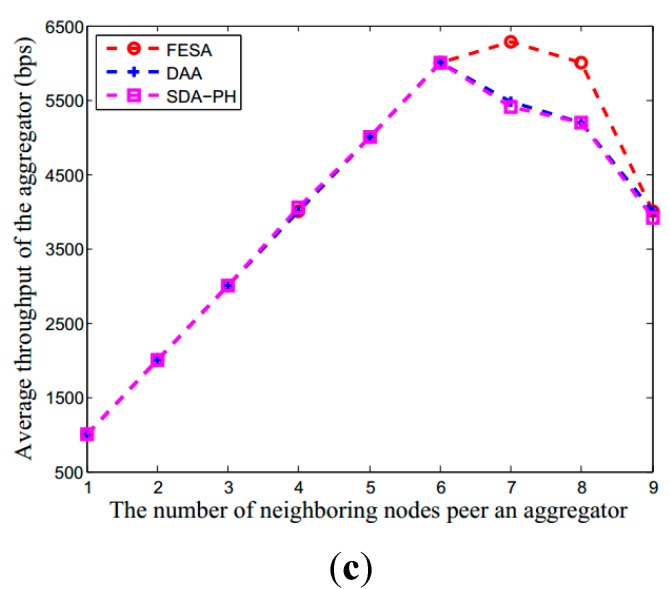
The performance in aggregator under DAA, SDA-PH and our scheme. Although it is more complex using FHE, the performance of our scheme is also better than other two schemes. (**a**) The average delay of aggregator; (**b**) The average delivery ratios of the aggregator; (**c**) The average throughput of the aggregator. In (**b**), when there are less than seven neighboring nodes forward data to an aggregator at the same time, the average data delivery ratios of the aggregator are close to 1.

Compared to the hop-by-hop encryption used in DAA and the privacy homomorphic encryption in SDA-PH, our scheme uses the FHE scheme to encrypt sensor data and achieve data confidentiality. Figure 5 illustrates the performance of these three protocols under different numbers of neighboring nodes of per aggregator, including transmission delay, delivery radios and throughput. The comparison result for average delays of the aggregator is SDA-PH < FESA < DAA. Since FESA increases part of the communication overhead using FHE, the average delivery ratios and the average throughput of the aggregator in FESA are better than the other two schemes, especially when there are more than six neighboring nodes for one aggregator.

#### 6.2.2. Communication Overhead of Network

The algorithm has a message overhead of 4 bytes per data packet as opposed to two MAC of 4 bytes each in DAA. Let *α* represent the number of data packets generated by legitimate nodes, and *β* represent the number of false data packets injected by up to *T* compromised nodes. Let *H* denote the average number of hops that a data packet travels in the network, and *H_f_* denote the average number of hops between two consecutive aggregators. Let *L_d_* denote the length of the data packet in FESA, then the data packet length in DAA is *L_d_* + 6. Let *D*_FESA_, *D*_DAA_ and *D*_SDA-PH_ denote the amount of data transmitted over a sensor network using FESA, DAA and SDA-PH, respectively. Therefore, *D*_FESA_, *D*_DAA_ and *D*_SDA-PH_ can be expressed as follows:

(8) $${\text{D}}_{\text{FESA}}{\text{ = L}}_{\text{d}}\text{ × }\left[\left(\text{α × H}\right)\text{ + }\left({\text{β × H}}_{\text{f}}\right)\right]{\text{+T × L}}_{\text{d}}\text{ × }\left(\text{α + β}\right)\text{+T × }\frac{\text{4}}{\text{T + 1}}\text{ × }\left(\text{α + β}\right)\text{ bytes}$$

(9) $${\text{D}}_{\text{DAA}}\text{ = }\left({\text{L}}_{\text{d}}\text{ + 6}\right)\text{ × }\left[\left(\text{α × H}\right)\text{ + }\left({\text{β × H}}_{\text{f}}\right)\right]\text{ + T × }\left({\text{L}}_{\text{d}}\text{ + 6}\right)\text{ × }\left(\text{α + β}\right)\text{ + T × }\frac{\text{4}}{\text{T+1}}\text{ × }\left(\text{α + β}\right)\text{ bytes}$$

(10) $${\text{D}}_{\text{SDA-PH}}\text{ = }\left({\text{L}}_{\text{d}}\;\text{- 8}\right)\text{ × H × }\left(\text{α + β}\right)\text{ bytes }$$

**Figure 6:**
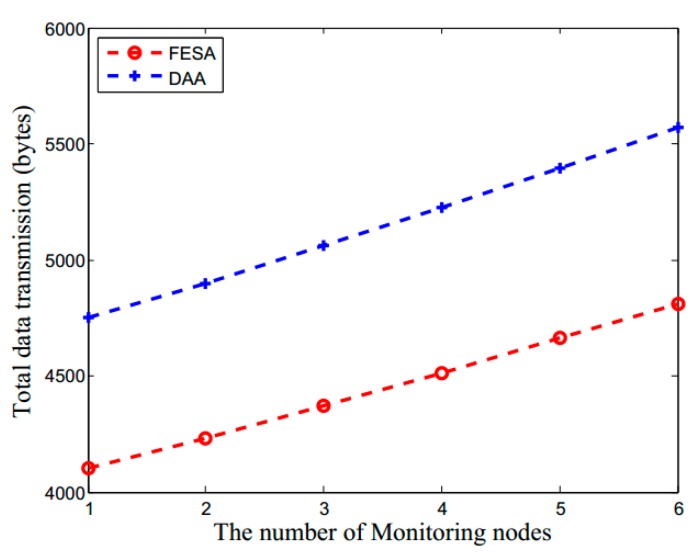
The impact of monitoring nodes on the communication overhead for FESA and DAA.

Although the value of *T* is nothing to do with the packet size, it can affect the number MAC transmissions between aggregators and monitoring nodes during data aggregation. Substituting *H* = 100 and *L_d_* = 38 in Equation (8), Figure 6 illustrates the effect of monitoring nodes to the total data transmission in FESA and DAA. As the number of monitoring nodes increases, the total data transmission in the network also increases. From Figure 6, we can see that DAA has a much more data transmission than FESA mainly because the data packets forwarded in DAA which contain both plaintext and ciphertext are larger than FESA.

Compared to no data aggregation protocols, the schemes using aggregators to aggregate receiving data can effectively reduce the data redundancy.

**Figure 7:**
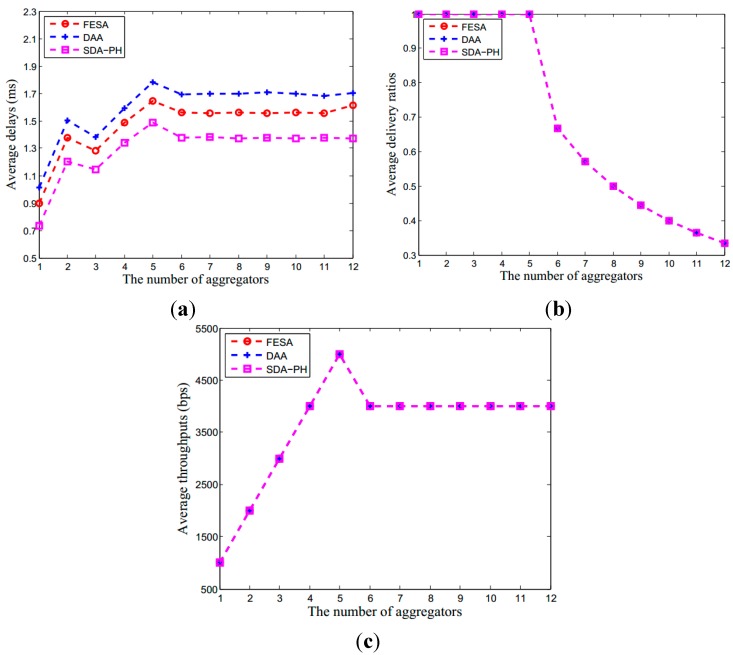
The entire network performance under DAA, SDA-PH and our scheme. Although using FHE increases the communication overhead, the performance of our scheme is still approaching to the other two schemes. (**a**) The average delay of the entire network; (**b**) The average delivery ratios of the entire network; (**c**) The average throughput of the entire network. In (**b**), when there are less than six aggregators forward data to the base station at the same time, the data delivery ratios are close to 1.

Therefore, in secure data aggregation protocols, the number of aggregators in the network affects the performance of the whole network. In this simulation scenario, we assume no false data exists. Figure 7 illustrates the performance of these three protocols under different number of aggregators, including transmission delay, delivery radios and throughput. The comparison result for average delays of the aggregator is SDA-PH < FESA < DAA. Since FESA increases part of the communication overhead using FHE, the average delivery ratios and the average throughputs of these three protocols are approximate.

**Figure 8:**
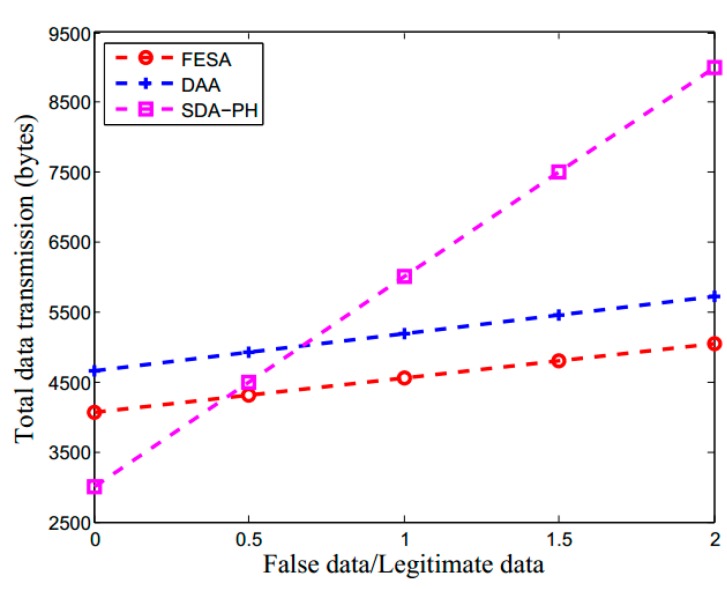
The total data transmission for FESA, DAA and SDA-PH, where in FESA and DAA detect false data during data aggregation and forwarding while SDA-PH detects only in BS.

Figure 8 shows the total data transmission *versus* *β*/*α* for FESA, DAA and SDA-HP. The total data transmission in the network is shown as a function of average number of hops between aggregators and the ratio of false data to legitimate data. Figure 8 shows that, as the ratio of false data to legitimate data increases, the total data transmission increases. Moreover, under the same conditions, compared to SDA-PH and DAA, FESA scheme has less total data transmission, which leads to the less energy consumption of sensor nodes. The reason is that each data packet of DAA is larger than FESA, thus more data are transmitted in the network. SDA-PH scheme detects false data only in the BS, therefore, data would not be detected until it transmitted into BS. In this condition, when more sensor nodes are compromised, FESA can quickly detect false data than SDA-PH, which results in the reduction of data transmission and energy consumption in the whole network.

## 7. Conclusions

In wireless sensor networks, an attacker can inject false data to damage the network data integrity by utilizing a compromised node. Existing researches did not combine data aggregation, data confidentiality, data integrity and false data detection together well. This paper proposes FESA, secure data aggregation with fully homomorphic encryption in large-scale wireless sensor networks. FESA can effectively reduce the network overhead while satisfying the above requirements. Compared to the existing technologies, our scheme can ensure the data confidentiality and integrity during data aggregation process and forwarding process, and also detect the false data as early as possible, leading to reduction of communication overhead and hence less energy consumption, thus prolong the life of the sensor nodes and networks. In our future work, we plan on investigating the further study of the performance of WSNs and the multiple applications of FHE such as in a VANET environment [31], and then lead to more secure and efficient data processing.
